# Reading Amount and Reading Strategy as Mediators of the Effects of Intrinsic and Extrinsic Reading Motivation on Reading Achievement

**DOI:** 10.3389/fpsyg.2020.586346

**Published:** 2020-10-27

**Authors:** Xiaocheng Wang, Lina Jia, Yuanying Jin

**Affiliations:** ^1^Department of Education, School of Humanities, Jiangnan University, Wuxi, China; ^2^Department of Education, School of Humanities, Sejong University, Seoul, South Korea

**Keywords:** intrinsic reading motivation, extrinsic reading motivation, reading amount, reading strategy, reading achievement, mediator

## Abstract

This study was conducted to examine the roles of reading amount and reading strategy as mediators of the effects of intrinsic and extrinsic reading motivation on reading achievement. A sample of 522 7th–9th graders from two public schools in Eastern China participated in the study and completed the questionnaires. The confirmatory factor analyses showed that Curiosity, Involvement, and Challenge as dimensions of intrinsic reading motivation and Recognition, Grades, and Competition as dimensions of extrinsic reading motivation represented reading motivation well in this Chinese sample population. Structural equation modeling analyses showed that intrinsic reading motivation had a positive direct effect on reading achievement, whereas extrinsic reading motivation exerted a negative direct effect on reading achievement. Both intrinsic and extrinsic reading motivation positively predicted reading strategy; however, only intrinsic reading motivation was positively correlated with reading amount. Neither reading amount nor reading strategy mediated the effects of intrinsic and extrinsic reading motivation on reading achievement. The implications of these findings for literacy research and instruction are discussed.

## Introduction

Reading proficiency is an indispensable competence (De Naeghel et al., 2012). The ability to read is an important prerequisite for learning; deficits in reading ability have considerable consequences for the acquisition of other necessary skills (Kirsch et al., 2002). Therefore, factors that facilitate development of reading competence are important to both teachers and researchers. In addition to cognitive factors, such as word recognition, strategy use, and prior knowledge, which are thought to mainly affect students’ reading competence, researchers have identified affective factors such as motivation (Baker and Wigfield, 1999; Guthrie and Wigfield, 2000). Even the most capable and skillful readers may choose not to read if they lack motivation, which ultimately decreases their reading proficiency (Stanovich, 1986).

Reading motivation is significantly correlated with various indicators of reading achievement (Baker and Wigfield, 1999; Guthrie et al., 1999). In particular, evidence suggests that intrinsic reading motivation positively predicts reading achievement, whereas extrinsic reading motivation is a non-significant or negative predictor (e.g., Andreassen and Bråten, 2010; Becker et al., 2010; see Schiefele et al., 2012 for an overview). Theoretically, the relationship between reading motivation and reading achievement is hypothesized to be mediated through reading amount (Wang and Guthrie, 2004; Schaffner et al., 2013) or reading strategy (Lim, 2013a,b). In other words, students with higher reading motivation levels are more likely to read frequently or to use diverse strategies while reading, both of which positively affect reading achievement.

However, direct empirical evidence for the relationship among intrinsic/extrinsic reading motivation, reading amount, reading strategy, and reading achievement is scarce. Notably, research on the mediating roles of reading amount or reading strategy is lacking in a Chinese sample. While motivational problems seem to be universal across cultural groups, it is unclear whether cultural differences have potential moderating effects on the relationships between reading motivation and other reading-related factors or whether the relationships observed in other cultures also apply in the Chinese cultural context. Therefore, the current study was conducted to examine the mediating effects of both reading amount and reading strategy on the relationship between intrinsic/extrinsic reading motivation and reading achievement in a Chinese sample.

### Conceptualization and Measurement of Reading Motivation

Reading motivation has been described as an individual’s subjective reasons for reading (Schiefele et al., 2012; Conradi et al., 2014). For example, a student may be motivated to read because he or she has a personal interest in a particular topic. Alternatively, the student’s motivation to read may arise from external incentives, such as the desire to obtain satisfactory grades in school or to gain recognition from others. These differing reasons and incentives are usually subsumed under two categories: intrinsic and extrinsic reading motivation (Wigfield and Guthrie, 1997; Unrau and Schlackman, 2006; Schiefele et al., 2012), which are theoretically distinct (Deci and Ryan, 1985; Ryan and Deci, 2000). *Intrinsic reading motivation* refers to the willingness to read because reading is perceived as rewarding or satisfying (Schiefele et al., 2012, Schaffner et al., 2013). This motivation usually arises from an individual’s personal interest in a particular activity or topic and is satisfied by pursuing that activity or topic (Unrau and Schlackman, 2006). Conversely, *extrinsic reading motivation* refers to reading because of external demands and values as opposed to reading for its own sake (Ryan and Deci, 2000). Extrinsically motivated reading tends to be driven by expected consequences, such as achieving positive outcomes or avoiding negative ones (Wigfield and Guthrie, 1997).

Various constructs of reading motivation have been examined to determine their relation to indicators of reading achievement. However, many have focused on only one or two dimensions of reading motivation, such as *attitude* (McKenna et al., 2012; Conradi et al., 2013), *self-efficacy* (Henk and Melnick, 1995; Henk et al., 2012), *self-concept* (Chapman and Tunmer, 1995), or the combination of *self-concept* and *value of reading* (Gambrell et al., 1996; Malloy et al., 2013). It is now well-established that motivation is a multidimensional concept that encompasses several interrelated constructs such as values, goals for achievement, and beliefs (Baker and Wigfield, 1999; Schiefele and Schaffner, 2016). Measuring only one or two dimensions is not sufficient to obtain a fuller understanding of students’ motivation to read. In line with this approach, Wigfield (1997); Wigfield and Guthrie (1997), and Baker and Wigfield (1999) developed the Motivation for Reading Questionnaire (MRQ), which is considered the most well-established and comprehensive instrument for measuring reading motivation (De Naeghel et al., 2012; Schiefele et al., 2012; Schiefele and Schaffner, 2016). The MRQ was developed based on various motivation theories, including self-efficacy theory (Bandura, 1997), self-determination theory (Ryan and Deci, 2002), and expectancy-value theory (Wigfield and Eccles, 2000). The MRQ proposes 11 constructs for reading motivation, which can be grouped into the following higher-order categories: *competence beliefs* (Self-efficacy, Challenge, and Work avoidance), *intrinsic reading motivation* (Curiosity, Involvement, and Importance), *extrinsic reading motivation* (Recognition, Grades, and Competition), and *social motivation* (Social and Compliance).

However, researchers only partially agree on which dimensions best constitute the intrinsic and extrinsic reading motivation constructs. In applying the MRQ to study reading motivation, researchers have used different composites of intrinsic and extrinsic reading motivation (Table 1). For example, some studies have used only Curiosity and Involvement to represent intrinsic reading motivation (e.g., Schiefele and Schaffner, 2016), while others have used Curiosity, Involvement, and Challenge as core dimensions of intrinsic reading motivation (e.g., Cox and Guthrie, 2001; Wang and Guthrie, 2004; Unrau and Schlackman, 2006; Logan et al., 2011). Furthermore, while some studies have used only Competition and Recognition as dimensions of extrinsic reading motivation (e.g., Schiefele and Löweke, 2017), others have regarded social motivation, which includes Social and Compliance, as components of extrinsic reading motivation in addition to Recognition, Grades, and Competition (e.g., Wang and Guthrie, 2004; Unrau and Schlackman, 2006; McGeown et al., 2016). Since different dimensions of reading motivation might exert different effects on other reading-related factors such as reading competence and reading strategies, researchers (e.g., Schiefele et al., 2012) have suggested that reaching a consensus on the definition and dimensions of reading motivation should be a high-priority task in future research.

**TABLE 1:** Comparison of reading motivation scales concerning intrinsic and extrinsic reading motivation.

	Wigfield and Guthrie, 1997; Baker and Wigfield, 1999: *Motivation for Reading Questionnaire*	Scales that correspond to the Motivation for Reading Questionnaire					
	
		Schiefele and Schaffner, 2016	Schiefele and Löweke, 2017	Guthrie et al., 1999; Cox and Guthrie, 2001	Logan et al., 2011	Schiefele et al., 2012	Wang and Guthrie, 2004; Unrau and Schlackman, 2006; McGeown et al., 2016
Intrinsic motivation	Curiosity	Curiosity	Curiosity	Curiosity	Curiosity	Curiosity	Curiosity
	Involvement	Involvement	Involvement	Involvement	Involvement	Involvement	Involvement
	Importance			Challenge	Challenge		Challenge
Extrinsic motivation	Recognition	Recognition	Recognition	Recognition	–	Recognition	Recognition
	Grades	Grades	Competition	Competition		Grades	Grades
	Competition	Competition				Competition	Competition
						Compliance	Compliance
							Social

### Reading Motivation and Reading Achievement

Reading motivation is significantly associated with various indicators of reading achievement (Baker and Wigfield, 1999; Guthrie et al., 1999, 2007; Unrau and Schlackman, 2006; Taboada et al., 2009). For example, Guthrie et al. (1999) showed that after controlling for variables including past achievement, socioeconomic status, self-efficacy, and reading amount, reading motivation significantly explained varying degrees of text comprehension among 10th graders. Evidence suggests that intrinsic reading motivation significantly positively affects various aspects of reading achievement, such as text comprehension, word recognition, and world knowledge, whereas extrinsic reading motivation has been non-significantly or even negatively associated with reading achievement (e.g., Cipielewski and Stanovich, 1992; Guthrie et al., 1999; Wang and Guthrie, 2004; Unrau and Schlackman, 2006; Taboada et al., 2009; Andreassen and Bråten, 2010; Becker et al., 2010; Lin et al., 2012; Schiefele et al., 2012).

Notably, studies that have used a single, composite measure of reading motivation (including both intrinsic and extrinsic reading motivation) have failed to show a significant association between the robust reading motivation construct and text comprehension (e.g., Guthrie et al., 1999; Cox and Guthrie, 2001). Given the theoretical distinctions between intrinsic and extrinsic reading motivation and the evidence showing that the two constructs exert opposite effects on reading achievement, using composite measures of reading motivation appears to neutralize the effect and thus seems inappropriate. In addition, intrinsic and extrinsic reading motivation are highly correlated, which induces a negative spurious effect of intrinsic reading motivation and a positive spurious effect of extrinsic reading motivation (cf. Wang and Guthrie, 2004). In other words, strong negative contributions of extrinsic reading motivation and strong positive contributions of intrinsic reading motivation occurred when both variables were simultaneously tested as predictors of reading achievement (Schiefele and Löweke, 2017). Arguably, intrinsic and extrinsic reading motivation should not be studied in isolation (Schiefele et al., 2012).

### The Mediating Role of Reading Amount

Analysis of mediating variables is important for investigating the causal relationship between reading motivation and reading achievement (Guthrie et al., 2012). The most important potential mediator discussed in previous research is reading amount—more precisely, the amount and frequency of reading for various purposes (Guthrie et al., 1999; Becker et al., 2010; Schiefele et al., 2012). As Guthrie et al. (1999) argued, “one of the major contributions of motivation to text comprehension is that motivation increases reading amount, which then increases text comprehension” (p. 250). This argument is based on evidence that reading motivation predicts reading amount (Wigfield, 1997; Wigfield and Guthrie, 1997; Guthrie et al., 1999; Cox and Guthrie, 2001) and that reading amount predicts text comprehension (Cipielewski and Stanovich, 1992; Cunningham and Stanovich, 1997; Cox and Guthrie, 2001; Schiefele et al., 2012).

On the one hand, reading motivation can lead to increased reading. For example, Wigfield and Guthrie (1997) reported that students with higher motivation levels read three times more frequently than do those with lower motivation levels. However, previous findings on the relative contributions of different reading motivation dimensions to reading amount were inconclusive. While some researchers have reported largely positive correlations between extrinsic reading motivation and the amount of reading for enjoyment, i.e., voluntary reading or reading for pleasure (e.g., Wigfield and Guthrie, 1997; Baker and Wigfield, 1999; Guthrie et al., 1999), others have found a weak negative correlation between these two variables (e.g., Becker et al., 2010). Additionally, some studies have shown a diminished positive effect, or even a negative effect, of extrinsic reading motivation on reading amount (e.g., Wang and Guthrie, 2004; Lau, 2009; Schaffner et al., 2013) when controlling for other relevant predictors such as prior reading achievement, intrinsic reading motivation, and reading efficacy. Generally, past research has consistently shown that intrinsic reading motivation often relates more strongly to the amount of reading for enjoyment than does extrinsic reading motivation (Baker and Wigfield, 1999; Wang and Guthrie, 2004; Lau, 2009; Becker et al., 2010).

On the other hand, reading amount is highly predictive of various indicators of reading achievement such as text comprehension, word recognition, and reading skills (Anderson et al., 1988; Cipielewski and Stanovich, 1992; Cunningham and Stanovich, 1997; Wigfield and Guthrie, 1997; Guthrie et al., 1999; Schiefele et al., 2012). For example, Cunningham and Stanovich (1997) estimated that 23% of the long-term growth in reading comprehension between the 5th and 10th graders was due to reading amount. Other evidence has shown that the amount of reading, whether for school or personal enjoyment, predicts the level of reading comprehension (e.g., Guthrie et al., 1999; Schiefele et al., 2012). Broad and frequent reading likely leads to desirable reading-related outcomes, such as vocabulary growth, automatization of basic reading processes (Schiefele et al., 2012; Stutz et al., 2016), increased use of reading strategies (Guthrie et al., 1999; Wigfield et al., 2008), and new topic knowledge (McNamara and Kintsch, 1996), all of which ultimately positively affect reading proficiency.

Although significant correlations between reading motivation and reading amount and between reading amount and reading achievement have been previously evidenced, there is little direct empirical evidence on the role of reading amount in mediating the effects of intrinsic/extrinsic reading motivation on reading achievement (for exceptions, see Wang and Guthrie, 2004; Becker et al., 2010; Schaffner et al., 2013). Furthermore, research has thus far been inconclusive on the mediating effects of reading amount. While some studies have found that reading amount does not significantly contribute to reading comprehension after controlling for both intrinsic and extrinsic reading motivation and is thus ineffective as a mediator of motivational effects (e.g., Wang and Guthrie, 2004; Becker et al., 2010), others have reported that reading amount fully mediates the positive effect of intrinsic reading motivation on reading comprehension and partially mediates the negative effect of extrinsic reading motivation on reading comprehension (e.g., Schaffner et al., 2013).

### The Mediating Role of Reading Strategy

Reading strategy is another important mediator assumed to mediate motivational effects on reading achievement. A reading strategy is a set of effort-consuming, potentially conscious and controllable mental or behavioral activities that can help students achieve cognitive purpose during reading (Flavell et al., 1993). Motivation is suggested to help activate cognitive processes, which can in turn affect achievement (Pintrich, 2003; Wigfield et al., 2006). In line with this assumption, a positive association between reading motivation and reading strategy (Guthrie et al., 1996; Guthrie and Wigfield, 2000; Cox and Guthrie, 2001; Lim, 2013a) and the way in which reading strategy affects reading achievement (Baker and Brown, 1984; Pokay and Blumenfeld, 1990; Lau and Chan, 2003) have been widely demonstrated.

Previous findings have shown moderate to high associations between reading motivation and reading strategy (e.g., Guthrie et al., 1996; Cox and Guthrie, 2001; Lau and Chan, 2003). As Paris et al. (1994) argued, the use of reading strategy incorporates both skill and will. Although many students may learn strategies, only motivated students will actually use them (Lau and Chan, 2003). Interestingly, whereas intrinsic reading motivation has been reported to be positively correlated with diverse reading strategies (e.g., Pintrich and De Groot, 1990; Cox and Guthrie, 2001; Lau and Chan, 2003), the correlation between extrinsic reading motivation and reading strategy remains ambiguous, as some studies have revealed non-significant effects of extrinsic reading motivation on reading strategy (e.g., Lau and Chan, 2003; Law, 2009; Andreassen and Bråten, 2010). Furthermore, regarding the level of reading strategy, intrinsically motivated students are assumed to comprehend text at deeper levels and are likely to use various strategies to achieve their goals (Pokay and Blumenfeld, 1990; Guthrie et al., 1999; Vansteenkiste et al., 2006), whereas extrinsically motivated students are assumed to use surface-level strategies, such as memorization and rehearsal (Schaffner and Schiefele, 2007), and to stop reading if the external rewards disappear (Deci and Ryan, 2000).

Reading strategy is also considered an important factor affecting text comprehension and reading achievement (Baker and Brown, 1984; Artelt et al., 2001; Lau and Chan, 2003). One significant characteristic of good readers is the knowledge and ability to choose and apply a repertoire of cognitive and metacognitive strategies during reading (Lau, 2017). As Patrick et al. (1999) argued, strategies used during the reading process, such as rehearsing, organizing, and elaborating meaning, are correlated with a mastery goal orientation, which reflects a commitment to understanding texts as deeply as possible. Consequently, many intervention programs have been developed to improve students’ reading comprehension through direct strategy instruction (e.g., Pressley et al., 1992; Pressley and Afflerbach, 1995). Despite this evidence, however, In Chinese language teaching, reading strategy instruction has not been emphasized. Chinese language teachers tend to teach reading indirectly by explaining prescribed texts (Tse et al., 1995; Lau, 2007, 2017). Thus, exploring the effect of strategy use on Chinese students’ reading achievement may have important implications for developing new approaches in Chinese reading instruction.

Based on findings that clearly support the effects of reading motivation on reading achievement and the close associations between reading strategy and motivational factors, researchers have suggested that cognitive factors, such as reading strategy, must be analyzed alongside motivational factors to better understand reading achievement (Guthrie et al., 1999; Guthrie and Wigfield, 2000; Lau and Chan, 2003). However, most previous studies have examined the contributions of either motivational or cognitive factors to reading achievement, and the association between reading strategy and extrinsic reading motivation has not been well studied (see an overview by Schiefele et al., 2012). Even fewer studies have examined the structural relationship linking both intrinsic and extrinsic reading motivation, reading strategy, and reading achievement (for an exception, see Lim, 2013a).

### Reading Motivation Among Chinese Students

Influenced by the Confucian heritage culture, traditional Chinese language instruction largely follows a teacher-centered and didactic approach (Ho, 2001; Lau and Chan, 2003; Hu, 2004). In a typical Chinese language class, the teacher explains the background knowledge, vocabulary, content, and rhetorical usage of each text in great detail following prescribed procedures (Han, 2000; Lau, 2017). Students only need to follow instructions and answer questions passively, relying on recitation and practice to memorize the knowledge and standardized explanations of the texts provided by their teachers (Tse et al., 1995; Hu, 2004). While curriculum reform has made this teaching approach less prevalent in current Chinese language classrooms, it remains one of the major reading instruction approaches in Mainland China (Cui, 2015), probably driven by the competitive examination system. Although this type of instructional approach is considered to be helpful to establish students’ foundation in reading (Lau, 2007, 2017), it clashes directly with adolescents’ increasing self-consciousness and need for autonomy (Eccles and Midgley, 1989). Consequently, students may perceive Chinese language classrooms as boring and find it difficult to develop internal interest in reading (Lau, 2007).

For example, although Chinese students ranked first in reading achievement on the Program for International Student Assessment (OECD, 2019), they have exhibited problems regarding reading motivation. Li (2014) reported that nearly 50% of Chinese adolescents read less than 3 h per week and 25% were unaccustomed to reading in their leisure time. Furthermore, although most Chinese adolescents consider reading as either very important or important, only 31.7% said that they actually like reading (Liu, 2015). Despite these figures, little is known about the reading motivations of Chinese students. Even fewer studies have investigated the causal relationship between reading motivation and other reading-related factors, such as reading behaviors and reading achievement. Most importantly, researchers have not explored the extent to which patterns observed in the Chinese culture differ from those of other cultures.

Compared to Western culture, Chinese culture is characterized as a collective one with an emphasis on human interdependence; hence, individuals are taught to be primarily concerned with the perspectives and feelings of others (Hong, 2001). Thus, social motivation seems to be particularly important for Chinese students who are socialized under a collectivistic culture (Yang, 1997) with teachers exerting a strong influence on Chinese students’ motivation levels (Lau and Chan, 2003; Lau, 2009; Huang et al., 2014). Moreover, given the highly competitive learning environment in Chinese societies and the importance of pursuing achievement in the Confucian tradition, some researchers (e.g., Shih, 2005; Lau and Lee, 2008) have suggested that extrinsic reading motivation, posited as a negative motivational orientation in Western theories, could be a positive source of motivation for Chinese students. Additionally, since the Chinese language test (e.g., midterm or final term examination) generally focuses on students’ memorization of background knowledge, vocabulary, content, and rhetorical usage of the prescribed texts in textbooks, and strategy instruction is not emphasized in Chinese language classrooms, it remains unclear whether the mediating effects of reading amount or reading strategy in the relationship between reading motivation and reading achievement as indicated in Western studies also apply to a Chinese context.

### The Present Study

**FIGURE 1:**
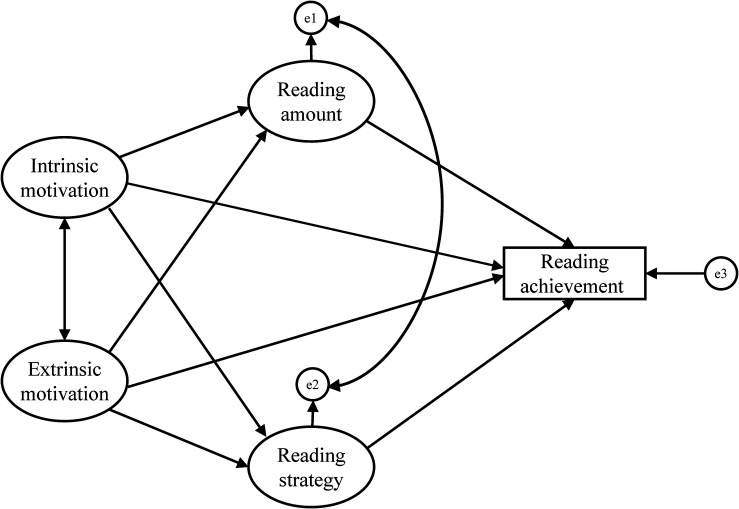
Hypothesized theoretical model.

Given the mixed evidence on reading amount as an explanatory mechanism in the relations between intrinsic/extrinsic reading motivation and reading achievement, and since previous research has not yet simultaneously addressed the role of reading strategy as a mediator of motivational effects on reading achievement (Schiefele et al., 2012), the present study further examines this issue. Based on the literature review, a theoretical structural model (Figure 1) was proposed for the present study that describes the relationships among intrinsic reading motivation, extrinsic reading motivation, reading amount, reading strategy, and reading achievement. Reading amount and reading strategy were permitted to be correlated with each other in the theoretical model since previous findings have indicated a close relationship between these two variables (e.g., Guthrie et al., 1999; Cox and Guthrie, 2001). In addition, as the dimensions of intrinsic and extrinsic reading motivation remain ambiguous, we first validated the abbreviated Chinese version of the MRQ that measures dimensions of intrinsic and extrinsic reading motivation. This was especially important given Schiefele et al.’s (2012) finding that the clarification of the dimensions of intrinsic and extrinsic reading motivation plays a dominant role in determining the relations of reading motivation to other reading-related variables. Specifically, the present study addresses the following two research questions.

**Question 1:** What is the factor structure of Chinese students’ intrinsic and extrinsic reading motivation?
**Question 2:** Does reading amount and reading strategy mediate the relationships between intrinsic/extrinsic reading motivation and reading achievement?

## Materials and Methods

### Participants

The research sample was recruited from two public schools in Eastern China. The same literacy curriculum was taught in both schools due to the consistent implementation of a national curriculum across Mainland China. After obtaining consent from the schools, the researchers, with assistance from individual classroom teachers, administered the questionnaires during regularly scheduled class periods. Questionnaires that were below 90% complete and had large missing values in at least one of the instruments were excluded (*n* = 20, 3.7%), producing a total of 522 questionnaires for analysis. The final sample consisted of 258 (49.4%) boys and 264 (50.6%) girls, among whom 187 (35.8%) were 7th graders, 171 (32.8%) were 8th graders, and 164 (31.4%) were 9th graders. Except for only one student who was 17 years old, all of the students were between the ages of 12 and 15 years old (*M* = 13.30 years, *SD* = 0.97).

### Instruments

The participants completed a survey that comprised the MRQ, the Survey of Adolescent Reading Attitudes (SARA, McKenna et al., 2012, for concurrent validity testing), the reading amount inventory, and the Cognitive Strategy Questionnaire (CSQ, OECD, 2009). All of the instruments, apart from the reading amount inventory, were adopted from pre-existing instruments and translated into Mandarin Chinese. A back-translation procedure was conducted to ensure precise translation. To ensure that each item was interpreted as intended, five students, none of whom participated in the study, reviewed the translated versions. Based on the students’ feedback and discussion among researchers, further modifications were made to improve vague phrases and inappropriate or ambiguous expressions. This cross-cultural adaptation process confirmed the appropriateness and comprehensibility of the language in a Chinese cultural context.

#### Reading Motivation

Reading motivation was measured using the Chinese version of the MRQ; our earlier research (Wang and Jin, in press) demonstrated the factorial validity of the Chinese MRQ using confirmatory factor analyses (CFAs). Based on theoretical considerations and previous findings, we used an abbreviated version of the MRQ (Appendix A) to assess the following dimensions of reading motivation: Curiosity (5 items), Involvement (5 items), Challenge (5 items), Importance (2 items), Grades (4 items), Recognition (5 items), Competition (4 items), Social (7 items), and Compliance (3 items). These scales were individually attributed to components of intrinsic motivation and extrinsic motivation. To determine suitable intrinsic and extrinsic reading motivational dimensions for the Chinese sample, four two-factor solutions were specified (see the detailed discussion in the analysis section). The MRQ consisted of statements such as, “*I like to read about new things*,” for the students to rate using a Likert-type scale scored as 1 (*very different from me*), 2 (*a little different from me*), 3 (*a little like me*), or 4 (*a lot like me*). In all cases, higher scores indicated higher motivational levels.

#### Reading Attitudes

Considering the close relationship between reading motivation and reading attitudes (Schiefele et al., 2012), reading attitudes were measured to verify the concurrent validity of the Chinese MRQ. Because adolescents’ attitudes toward reading are not fixed but vary greatly by context, especially with regard to reading purpose and medium (McKenna et al., 2012), the SARA, which measures adolescents’ attitudes toward reading in different contexts, was selected as a criterion measure. The validated Chinese version of the SARA (Appendix B, see Wang and Jin, 2020) contains 15 items that vary along two dimensions: reading purpose (academic versus recreational) and medium (print versus digital). Accordingly, the items were divided into four subscales regarding attitudes toward academic print (5 items), academic digital (5 items), recreational print (5 items), and recreational digital (3 items) reading. Each item began with “*How do you feel about*…” and was rated on a Likert-type scale with responses ranging from 1 (*very bad*) to 6 (*very good*). The Chinese SARA had an internal-consistency reliability value of 0.85 for the total scale and ranged from 0.72 to 0.78 for the four subscales.

#### Reading Amount

Reading amount was measured based on the references of the Reading Activity Inventory (Guthrie et al., 1994) and the reading amount scale (Schaffner et al., 2013). Given the differential associations between reading motivation and reading for enjoyment and school-related reading (Cox and Guthrie, 2001; Wang and Guthrie, 2004; Schiefele et al., 2012) as well as evidence that the amount of reading for enjoyment contributes more strongly to reading achievement than does the amount of reading for school (Schiefele et al., 2012), using a composite measure of reading amount comprising both reading types seemed inappropriate. Therefore, this study used three questions to measure only the amount of reading for enjoyment (Appendix C). The first and third questions were adapted from Schaffner et al. (2013), asking students how many books they had read for interest during the previous month (1 = *0 books*; 2 = *1–2 books*; 3 = *3–4 books*; 4 = *more than 5 books*) and how long they usually spent reading a book without taking a break when reading for interest (1 = *5 min*; 2 = *15 min*; 3 = *30 min*; 4 = *60 min or more*). The second question was adapted from Guthrie et al. (1994), asking students how often they read for interest (1 = *almost never*; 2 = *once a month*; 3 = *once a week*; 4 = *almost every day*). To better understand students’ reading behavior, we asked them to write down the titles of books (up to a maximum of three) that they had recently read for interest. The Cronbach’s alpha of the scale was 0.66, similar to that of previous studies (e.g., Wang and Guthrie, 2004; Stutz et al., 2016).

#### Reading Strategy

Reading strategy was measured by the CSQ, which assesses the frequency of strategies used when reading. The CSQ (Appendix D) consists of 13 items divided into three dimensions: Memorization (4 items), Elaboration (4 items), and Control (5 items). These dimensions correspond closely to the reading strategy categories specified in Pressley and Afflerbach’s (1995) review of think-aloud studies. Memorization and Elaboration are two cognitive techniques. Memorization is the process of forming verbatim representations and storing them in memory through repetition, without moving beyond or transforming what is presented in the text (e.g., “*I try to memorize everything that is covered in the text*”). Elaboration is the process by which students transfer and integrate information by relating what they have learned to other contexts (e.g., “*I try to relate new information to prior knowledge acquired in other subjects*”). Control reflects a metacognitive aspect of students’ ability; it is used to assess or self-regulate the learning process and comprehension, ensuring that goals are achieved (e.g., “*I try to figure out which concepts I still haven’t really understood*”). Each question began with “*When I study, I*…” and was rated using a Likert-type scale, with responses ranging from 1 (*almost never*) to 4 (*almost always*). The Cronbach’s alpha of the total scale was 0.93. For the three subscales, the Cronbach’s alphas were 0.77 (Memorization), 0.83 (Elaboration), and 0.84 (Control).

#### Reading Achievement

Reading achievement refers to students’ grades in the midterm Chinese language test, which was implemented approximately 1 week before the survey administration. The test lasted for 2 h, focusing on knowledge of words, idioms, grammar rules and usage, and reading comprehension of classic and modern Chinese literature, and world literature. Students’ scores were graded on a scale of 0–100, and the mean score was 77.0. For statistical analysis, we transferred the raw scores to *Z* scores. To relate students’ test grades to their questionnaire responses for matching, the classroom teachers first numbered each student’s score and then the questionnaires. The questionnaires were administered to the students based on their numbers. For example, Paul (pseudonym) was given questionnaire No. 10 since his number in the school report card was 10.

## Analysis

**FIGURE 2:**
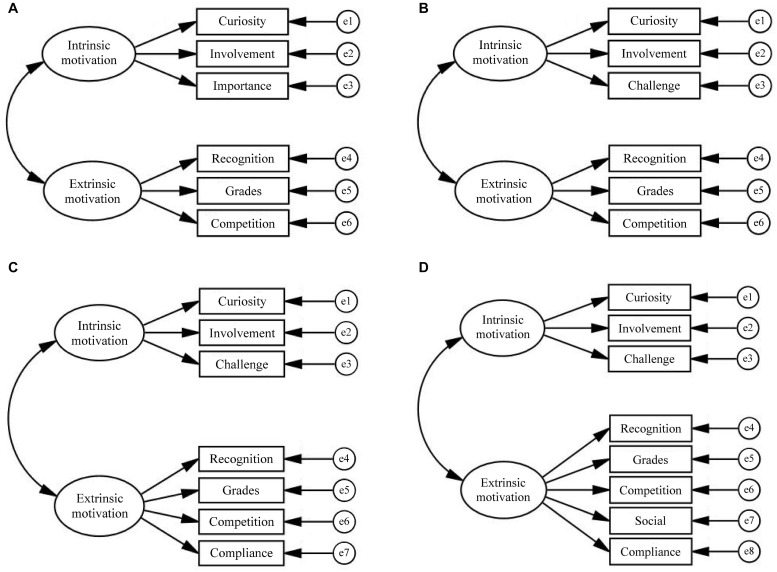
Hypothesized two-factor models. **(A)** Two-factor model A. **(B)** Two-factor model B. **(C)** Two-factor model C. **(D)** Two-factor model D.

All statistical analyses were conducted using SPSS 22.0 and Amos 22.0. First, missing data were handled using the expectation maximization algorithm. Skewness and kurtosis tests were conducted to examine the data distribution. For the first research question, we performed CFAs using the maximum likelihood estimation method in Amos 22.0. Based on theoretical and empirical rationales, CFAs were conducted on the MRQ items for four two-factor solutions: Models A, B, C, and D (Figure 2). Model A hypothesized that intrinsic reading motivation underpinned the three internal constructs of Curiosity, Involvement, and Importance, while extrinsic reading motivation underpinned the three external constructs of Recognition, Grades, and Competition, as the scale developers originally suggested (Wigfield and Guthrie, 1997; Baker and Wigfield, 1999). Given that many previous studies have regarded Challenge as a form of intrinsic reading motivation (e.g., Cox and Guthrie, 2001; Wang and Guthrie, 2004; Unrau and Schlackman, 2006; Logan et al., 2011) and that the findings of Guthrie et al. (1996) did not suggest Importance, Challenge was used in place of Importance in Model B to represent intrinsic reading motivation. Model C maintained the same intrinsic-motivation-factor constructs as Model B; however, it hypothesized that extrinsic reading motivation underpinned the four external constructs of Recognition, Grades, Competition, and Compliance (Schiefele et al., 2012). Based on Model C, Model D added Social as an additional construct of extrinsic reading motivation (Wang and Guthrie, 2004; Unrau and Schlackman, 2006). Self-efficacy was not included as a component of intrinsic reading motivation since it represents a theoretically independent construct (see also Guthrie et al., 1999; Schiefele et al., 2012). Using SPSS 22.0, Cronbach’s alphas were computed for the total and each subscale of the MRQ, and the bivariate correlations between the raw scores of the MRQ and SARA subscales were examined to further evaluate the construct validity of the MRQ.

For the second research question, we performed a structural equation modeling (SEM) analysis to examine the relationships among intrinsic reading motivation, extrinsic reading motivation, reading amount, reading strategy, and reading achievement. According to Kline (2015), an SEM analysis should proceed in two steps, testing the measurement and structural models. We first specified a full measurement model which included all of the latent variables and conducted a CFA to test the appropriateness of the model. We then specified a structural model to test the hypothesized mediation model. Because the mediation model has two mediator variables, Monte Carlo analyses were used to test whether the indirect effects of each mediator variable were statistically significant (Preacher and Selig, 2012). Monte Carlo confidence intervals that did not include zero suggested a 95% probability that the indirect effect was significant.

Because the chi-square (χ^2^) test is sensitive to sample size (Marsh et al., 1988), we used the comparative fit index (CFI), the Tucker–Lewis index (TLI), the root mean square error of approximation (RMSEA), and the standardized root mean square residual (SRMR) to assess the level of fit for both the measurement and structural models. An adequate and good model fit may be indicated by values greater than 0.90 and 0.95, respectively, on CFI and TLI, and by values lower than 0.08 and 0.06, respectively, on RMSEA and SRMR (Hu and Bentler, 1999). The Akaike Information Criterion (AIC) and Bayesian Information Criterion (BIC) were used to compare the non-nested models, with lower values indicating a better fit (Kline, 2015).

## Results

### Dimensionality of Intrinsic and Extrinsic Reading Motivation

**FIGURE 3:**
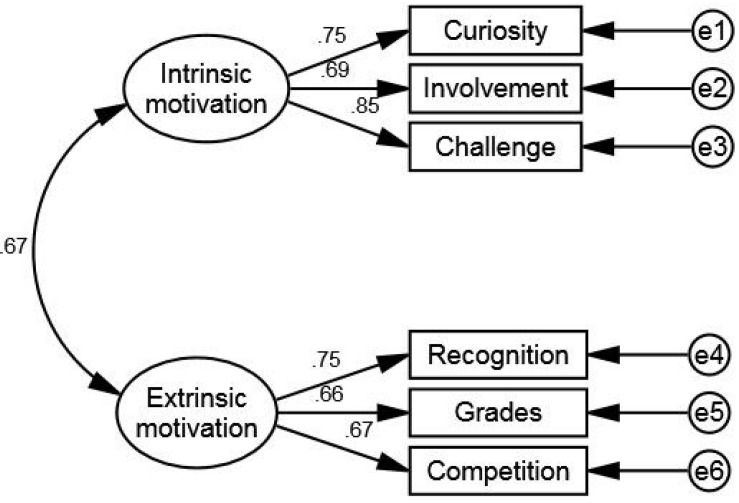
Two-factor model B for the abbreviated Chinese version of the MRQ.

The CFA results indicated that Model A did not fit the data well, as indicated by RMSEA, while Models B, C, and D had good levels of fit with the data (Table 2). Salient decreases in AIC and BIC were found in Model B (Model C vs. Model B: ΔAIC = 17.56, ΔBIC = 26.07; Model D vs. Model B: ΔAIC = 52.76, ΔBIC = 69.79), showing that Model B best explained the covariance among the motivational variables. Thus, Model B, which proposed Curiosity, Involvement, and Challenge as dimensions of intrinsic reading motivation, and Recognition, Grades, and Competition as dimensions of extrinsic reading motivation, was selected as the potential final model for use among Chinese students. The factor structure and standardized parameter estimates of Model B are presented in Figure 3. As can be seen, each item significantly loaded on its respective construct, with the factor loadings ranging from 0.66 (*p* < 0.001) to 0.85 (*p* < 0.001).

**TABLE 2:** Model fit indexes.

Models		χ^2^	*df*	CFI	TLI	RMSEA	SRMR	AIC	BIC
								
				Good: ≥0.95	Good: ≥0.95	Good: ≤0.06	Good: ≤0.06	The lower, the better	
				Acceptable: ≥0.90	Acceptable: ≥0.90	Acceptable: ≤0.08	Acceptable: ≤0.08		
A	IM: Curiosity, Involvement, Importance EM: Recognition, Grades, Competition	55.43***	8	0.947	0.900	0.107	0.046	81.43	136.78
B	IM: Curiosity, Involvement, Challenge EM: Recognition, Grades, Competition	29.70***	8	0.979	0.961	0.072	0.037	55.70	111.05
C	IM: Curiosity, Involvement, Challenge EM: Recognition, Grades, Competition, Compliance	43.26***	13	0.976	0.961	0.067	0.038	73.26	137.12
D	IM: Curiosity, Involvement, Challenge EM: Recognition, Grades, Competition, Compliance, Social	74.46***	19	0.965	0.948	0.075	0.038	108.46	180.84

IM, intrinsic motivation; EM, extrinsic motivation; CFI, comparative fit index; TLI, Tucker–Lewis index; RMSEA, root mean square error of approximation; SRMR, standardized root mean square residual; AIC, Akaike Information Criterion; BIC, Bayesian Information Criterion. ***p < 0.001.

The internal consistency coefficient for the whole scale was 0.81; the coefficients for the two subscales were 0.89 (intrinsic) and 0.88 (extrinsic). The validity of the Chinese MRQ was also supported by its significant correlations with the SARA as a criterion measure. Specifically, all MRQ dimensions were significantly associated with the SARA subscales (0.15 ≤ *r* ≤ 0.49, *p* < 0.01), except Challenge, which was uncorrelated with attitudes toward recreational digital reading (Table 3). Notably, intrinsic reading motivation was most strongly correlated with attitudes toward recreational print reading (*r* = 0.55, *p* < 0.01) while it was weakly correlated with attitudes toward recreational digital reading (*r* = 0.16, *p* < 0.01). Extrinsic reading motivation was more strongly correlated with attitudes toward academic reading in both print (*r* = 0.47, *p* < 0.01) and digital (*r* = 0.38, *p* < 0.01) settings.

**TABLE 3:** Correlation coefficients between reading motivation and reading attitudes.

	Intrinsic motivation				Extrinsic motivation			
		
	Curiosity	Involvement	Challenge	Total scale scores of intrinsic motivation	Recognition	Grades	Competition	Total scale scores of extrinsic motivation
Academic digital	0.36**	0.33**	0.36**	0.41**	0.33**	0.30**	0.29**	0.38**
Recreational digital	0.15**	0.18**	0.07	0.16**	0.22**	0.13**	0.21**	0.23**
Academic print	0.32**	0.28**	0.42**	0.40**	0.44**	0.35**	0.35**	0.47**
Recreational print	0.49**	0.42**	0.49**	0.55**	0.39**	0.24**	0.27**	0.37**
Total scale scores of reading attitudes	0.43**	0.40**	0.42**	0.49**	0.46**	0.33**	0.38**	0.48**

**p < 0.01.

### Descriptive Statistics and Intercorrelations

Table 4 presents the means and standard deviations of all variables and their intercorrelations. The mean scores of all motivation variables exceeded the midpoint of 2.50, showing that Chinese students characterized themselves as motivated readers with respect to all dimensions. Between the two subscales, Chinese students scored higher on intrinsic reading motivation (*M* = 3.29) than on extrinsic reading motivation (*M* = 2.88). Moreover, intrinsic reading motivation was positively correlated with reading amount (*r* = 0.34, *p* < 0.01), reading strategy (*r* = 0.59, *p* < 0.01), and reading achievement (*r* = 0.20, *p* < 0.01). For extrinsic reading motivation, while it was positively correlated with reading amount (*r* = 0.19, *p* < 0.01) and reading strategy (*r* = 0.52, *p* < 0.01), no correlation with reading achievement was found. Notably, as one of the dimensions of extrinsic reading motivation, Recognition was more strongly correlated with reading amount (*r* = 0.23, *p* < 0.01), reading strategy (*r* = 0.52, *p* < 0.01), and intrinsic reading motivation (*r* = 0.52, *p* < 0.01) than were the other two extrinsic motivation variables, i.e., Grades and Competition.

**TABLE 4:** Descriptive statistics, reliability coefficients, and intercorrelations among the measured variables.

Variables		Mean	Standard deviation	Cronbach’s alpha	1	2	3	4	5	6	7	8	9	10	11	12	13
(1)	Intrinsic motivation	3.29	0.48	0.89	–												
	(2) Curiosity	3.30	0.53	0.78	0.84**	–											
	(3) Involvement	3.21	0.52	0.77	0.76**	0.53**	–										
	(4) Challenge	3.23	0.58	0.81	0.88**	0.64**	0.58**	–									
(5)	Extrinsic motivation	2.88	0.56	0.88	0.52**	0.41**	0.40**	0.49**	–								
	(6) Recognition	2.93	0.67	0.86	0.52**	0.41**	0.39**	0.49**	0.84**	–							
	(7) Grades	2.80	0.68	0.73	0.32**	0.24**	0.26**	0.31**	0.73**	0.49**	–						
	(8) Competition	2.88	0.66	0.76	0.40**	0.33**	0.29**	0.38**	0.73**	0.46**	0.51**	–					
(9)	Reading amount	3.00	0.71	0.66	0.34**	0.31**	0.25**	0.29**	0.19**	0.23**	0.08	0.11*	–				
(10)	Reading strategy	3.02	0.60	0.93	0.59**	0.45**	0.43**	0.57**	0.52**	0.52**	0.33**	0.37**	0.20**	–			
	(11) Memorization	2.96	0.65	0.77	0.52**	0.39**	0.39**	0.51**	0.47**	0.48**	0.32**	0.31**	0.19**	0.91**	–		
	(12) Elaboration	3.01	0.67	0.83	0.53**	0.40**	0.38**	0.51**	0.48**	0.47**	0.30**	0.38**	0.16**	0.92**	0.74**	–	
	(13) Control	3.09	0.63	0.84	0.58**	0.45**	0.43**	0.56**	0.48**	0.50**	0.30**	0.33**	0.20**	0.93**	0.79**	0.80**	–
(14)	Reading achievement	–	–	–	0.20**	0.19**	0.17**	0.16**	0.02	0.08	–0.07	–0.03	0.13**	0.12**	0.07	0.08	0.17**

*p < 0.05, **p < 0.01.

### Test of the Hypothesized Model

Before examining the hypothesized structural model, we specified a full measurement model and conducted a CFA to test the appropriateness of this model. The CFA results indicated that the full measurement model had a high level of fit to the data: χ^2^(48) = 105.24, *p* < 0.001, CFI = 0.978, TLI = 0.970, RMSEA = 0.048, SRMR = 0.036. The latent factors were correlated with values ranging from 0.28 (*p* < 0.001) to 0.68 (*p* < 0.001). Each item significantly loaded on its respective construct, with values ranging from 0.50 (*p* < 0.001) to 0.92 (*p* < 0.001), demonstrating that the scales for measuring each construct had adequate convergent validity. The Cronbach’s alpha for each subscale further provided evidence that the measurement model was reliable and appropriate (Table 4). An SEM analysis was performed next.

**FIGURE 4:**
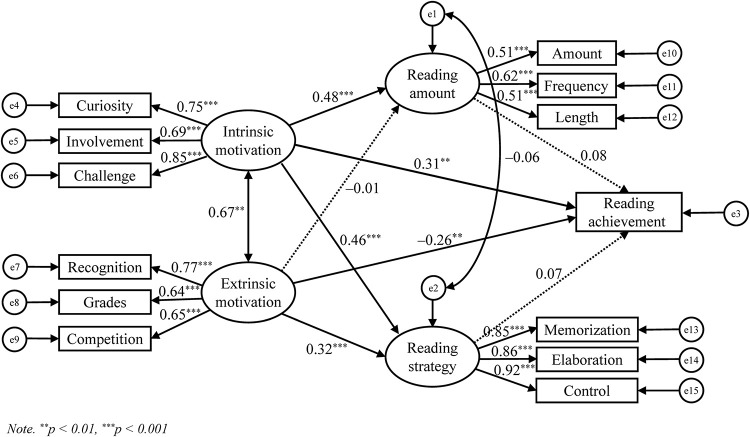
Model of relations among intrinsic reading motivation, extrinsic reading motivation, reading amount, reading strategy, and reading achievement.

The structural analysis results showed a high level of fit to the data: χ^2^(56) = 125.40, *p* < 0.001, CFI = 0.974, TLI = 0.963, RMSEA = 0.049, SRMR = 0.037. The structural model (Figure 4) showed that intrinsic reading motivation had a direct positive effect on reading achievement (β = 0.31, *p* < 0.01), while extrinsic reading motivation had a direct negative effect on reading achievement (β = −0.26, *p* < 0.01). Intrinsic reading motivation also strongly predicted reading amount (β = 0.48, *p* < 0.001) and reading strategy (β = 0.46, *p* < 0.001). Extrinsic reading motivation was uncorrelated with reading amount (β = −0.01, *p* = 0.93) but was positively correlated with reading strategy (β = 0.32, *p* < 0.001). Unexpectedly, neither reading amount (β = 0.08, *p* = 0.27) nor reading strategy (β = 0.07, *p* = 0.35) significantly explained the variance in reading achievement. The results of Monte Carlo analyses revealed that the indirect effects of intrinsic reading motivation on reading achievement were neither mediated by reading amount (95% CI [−0.081, 0.294]) nor by reading strategy (95% CI [−0.089, 0.283]). Similarly, extrinsic reading motivation was not indirectly correlated with reading achievement through the mediation role of reading amount (95% CI [−0.049, 0.054]) or reading strategy (95% CI [−0.051, 0.176]). Thus, both reading amount and reading strategy are ineffective as mediators of motivational effects on reading achievement.

## Discussion

This study was conducted to investigate the roles of reading amount and reading strategy as mediators of the contributions of intrinsic and extrinsic reading motivation on reading achievement. Given the existing debate about the dimensions of intrinsic and extrinsic reading motivation, we first validated the abbreviated Chinese MRQ to determine which dimensions best constituted intrinsic and extrinsic reading motivation among Chinese students.

### Dimensionality of Intrinsic and Extrinsic Reading Motivation

The CFA results showed that Model A did not adequately explain the data, as indicated by RMSEA. This result supported Guthrie et al.’s (1996) finding that did not suggest Importance as a dimension of intrinsic reading motivation. The CFA results also demonstrated that Model B was the best solution for measuring the intrinsic and extrinsic reading motivation of the Chinese student participants, further confirming the use of Curiosity, Involvement, and Challenge as dimensions of intrinsic reading motivation (Cox and Guthrie, 2001; Wang and Guthrie, 2004; Logan et al., 2011) and Recognition, Grades, and Competition as dimensions of extrinsic reading motivation (Wigfield and Guthrie, 1997; Baker and Wigfield, 1999; Schiefele and Schaffner, 2016). Additionally, in line with previous research (Wang and Guthrie, 2004; Unrau and Schlackman, 2006), intrinsic and extrinsic reading motivation were highly positively correlated, indicating that intrinsic and extrinsic incentives often simultaneously motivate reading, possibly to varying degrees (Schiefele et al., 2012).

Social motivation, incorporating Social and Compliance, seems less appropriate for measuring the extrinsic reading motivation of Chinese students. It should be noted that items on the Social scale may express both preference for and frequency of literacy practices within family and peer groups. Most items discussed specific reading-related activities in a social context, for example, “*I often read to my brother or my sister*.” However, because a student might read to his or her siblings either in response to parental expectations or because reading aloud is an enjoyable experience, inferring social motivations is difficult when the items do not address reasons for engaging in these activities (Schiefele et al., 2012; Schiefele and Schaffner, 2016). Furthermore, some Social items refer to personal preferences (e.g., “*My friends and I like to trade things to read*”), which may partially represent intrinsic motivation in reading.

As another indicator of social motivation, Compliance was more related to the reading demands made by schools and teachers; it addressed reading for school rather than for pleasure (e.g., “*I always do my reading work exactly as the teacher wants it*”). Our earlier research (Wang and Jin, in press) showed that social motivation, especially Compliance, was one of the dimensions most strongly endorsed by Chinese students, implying that Chinese students tend to be motivated to read because school curricula or teachers require it, in which case they tend to make the effort to finish every reading assignment exactly as the teacher has stipulated. Social motivation seems particularly important to Chinese students, who have been socialized within a collectivistic culture and are thus more likely to be socially oriented learners (Wang, 2001). According to the self-determination theory (Deci and Ryan, 1985; Ryan and Deci, 2000), extrinsic motivation has various levels, ranging from external regulation (completely controlled by external reinforcement) to integrated regulation (reflecting the internalization and integration of external values). When Chinese students fully identify with and internalize the values inherent in social relationships, conformity, and the respectful acceptance of advice from others, especially teachers, as emphasized by the Confucian tradition (Hong, 2001), this extrinsic motivation may explain some of the variance in intrinsic motivation. Thus, using social motivations as components of extrinsic reading motivation among Chinese students seems inappropriate.

Similarly, Recognition, which indicates the pleasure deriving from receiving recognition for success in reading, was proposed as one of the dimensions of extrinsic reading motivation. It is necessary to reconsider whether Recognition might represent some variance of social motivation or intrinsic motivation for Chinese students, due to the emphasis of the Chinese collective culture on human interdependence and the consideration of others’ perspectives and feelings (Hong, 2001). This view also finds support in the fact that Recognition was more strongly correlated with reading amount, reading strategy, and intrinsic reading motivation than were the other two extrinsic motivation variables. This result suggests that Chinese students may perceive Recognition differently from the other two extrinsic motivation variables.

The concurrent validity of the Chinese MRQ was supported by significant positive correlations among subscales with reading attitudes. The only exception was Challenge, which was not significantly correlated with attitudes toward recreational digital reading. This result was somewhat expected, as Chinese students perceived recreational digital reading as very different from the other three reading types (see Wang and Jin, 2020, for more details of this issue). This view could be further supported by the fact that attitudes toward recreational digital reading had the lowest correlations with both intrinsic and extrinsic reading motivation. Notably, intrinsic reading motivation was most highly correlated with attitudes toward recreational print reading while it was least strongly correlated with attitudes toward recreational digital reading. This result was consistent with previous findings that attitudes toward recreational print reading more strongly predicted reading achievement while attitudes toward recreational digital reading did not influence reading achievement (Lupo et al., 2017; Jang and Ryoo, 2019). In addition, extrinsic reading motivation was more strongly correlated with attitudes toward academic reading in both print and digital settings, probably due to the item contents of extrinsic reading motivation that were more related to academic, school reading contexts.

### Test of the Hypothesized Model

Structural equation modeling results suggested that intrinsic reading motivation contributed strongly positively to reading achievement, whereas extrinsic reading motivation directly negatively affected reading achievement. This result further evidences that intrinsic and extrinsic reading motivation are differently associated with reading achievement (Wang and Guthrie, 2004; Lau, 2009; Becker et al., 2010; Schiefele et al., 2012, Schaffner et al., 2013). When extrinsically motivated students read, they tend to focus on expected outcomes, such as meeting external demands or obtaining rewards, rather than learning from the texts or using deep-level strategies to overcome difficulties during reading (Wang and Guthrie, 2004; Hulleman et al., 2008; Schaffner et al., 2013). Focusing on the consequences of reading can reportedly lead students to use surface-level strategies, such as memorization and guessing (Elliott and Dweck, 1988; Wigfield et al., 2016), which negatively predict reading comprehension (Lim, 2013a). Consequently, the text comprehension process may be degraded. Notably, the observed zero-order correlation between intrinsic reading motivation and reading achievement was much lower than the path coefficient in the structural model, and the correlation between extrinsic reading motivation and reading achievement even became negative in the structural model. This effect might be related to the high correlation between intrinsic and extrinsic reading motivation (cf. Wang and Guthrie, 2004; Unrau and Schlackman, 2006; Schiefele et al., 2012; Schaffner et al., 2013). The same explanation applies to the discrepancy between findings from the correlational and SEM analyses pertaining to reading amount.

Furthermore, intrinsic reading motivation showed significant positive correlations with reading amount, whereas extrinsic reading motivation was uncorrelated with reading amount. This result was consistent with previous findings suggesting that intrinsic reading motivation enhances the amount of voluntary reading (Guthrie et al., 1999; Schaffner et al., 2013) and that extrinsic reading motivation non-significantly or negatively contributed to reading amount (Wang and Guthrie, 2004; Lau, 2009; Becker et al., 2010; Schiefele et al., 2012; Schaffner et al., 2013). As Schaffner et al. (2013) suggested, reading is largely a leisure-time activity; thus, it is more strongly intrinsically incentivized. Students exhibiting high levels of intrinsic reading motivation may be more likely to read widely and frequently in their spare time (Wigfield, 1997; Guthrie et al., 1999). In contrast, extrinsically motivated students may not consider reading for enjoyment as an effective way to increase their reading achievement in school (Schaffner et al., 2013). Such students are likely to read only when they have to, for example, to gain praise from others or achieve at higher levels. This approach leads to decreased reading for enjoyment and poorer reading skills (Becker et al., 2010).

Unexpectedly, the SEM results did not support the suggestion that the relationships between intrinsic/extrinsic reading motivation and reading achievement are mediated by reading amount. This result is in line with the findings from Wang and Guthrie (2004), which suggested that this result may pertain to the common link between reading amount and reading achievement being intrinsic reading motivation. In other words, students who had less intrinsic motivation spent less time reading and were less likely to be successful in text comprehension. Another possible explanation is that the contents of Chinese language test are more related to those from textbooks as well as teacher lectures, in which memorization of knowledge and standardized interpretations of texts (as explained by teachers) were emphasized. Thus, students’ amount of reading for enjoyment, which is generally regarded as “wasting time” (Hu, 2004) and is more related to reading materials considered as *light reading* (e.g., comics or novels, as indicated by the book titles students wrote down), did not seem to influence students’ academic literacy. It is necessary to use more objective measures, such as a standardized reading comprehension test, to further investigate the effect of reading amount on reading achievement. It is also worth exploring that whether the contribution of reading behavior to reading comprehension may not only depend on the amount or frequency of reading but also on the nature and the level of challenge of reading materials (Schiefele et al., 2012).

On the other hand, both intrinsic and extrinsic reading motivations were positively associated with reading strategy. This result confirmed previous findings suggesting that reading motivation, whether internal or external, provides an energizing and activating role for cognitive processes (Cox and Guthrie, 2001; Pintrich, 2003; Wigfield et al., 2006; Lim, 2013a). However, this result was inconsistent with previous studies that revealed a significant correlation between strategy use and intrinsic reading motivation, but not extrinsic reading motivation (Lau and Chan, 2003; Law, 2009; Andreassen and Bråten, 2010). This inconsistency may be due to the reading strategy construct used in this study that consisted of both surface- (Memorization) and deep-level (Elaboration, Control) strategies (see also Entwistle and McCune, 2004; Anmarkrud and Bråten, 2009; Lim, 2013a,b), which may have had different associations with other reading-related variables, such as intrinsic/extrinsic reading motivation and reading achievement. For example, research has indicated that extrinsically motivated students are more likely to use surface-level strategies (Schaffner and Schiefele, 2007), while intrinsically motivated students may apply deeper strategies (Lim, 2013a).

We could not confirm the mediating effect of reading strategy in the relationships between intrinsic/extrinsic reading motivation and reading achievement. To better understand this result, it is necessary to note that strategy instruction has not been emphasized in Chinese language classrooms, in which reading has been taught indirectly by explaining or interpreting an assigned text (Lau and Chan, 2003; Hu, 2004; Lau, 2009). In addition, the Chinese language test focuses on students’ knowledge of words, idioms, grammar rules and usage, and the ability to memorize standardized explanations of texts that provided by teachers. Thus, the positive effect of reading strategy on reading achievement might be diminished.

### Practical Implications

The present findings have important implications for teaching. First, the CFA results indicated that Challenge better represented intrinsic reading motivation of Chinese students than did Importance. Teachers may therefore consider using Challenge as an important indicator of intrinsic reading motivation of Chinese students, adopting reading materials with adequate challenging levels to engage students in reading.

Second, the SEM models highlighted the importance of intrinsic reading motivation, which will likely increase students’ reading amount, strengthen reading strategy, and improve reading achievement. Thus, interventions designed to enhance students’ reading motivation should focus on encouraging internal reasons for reading. This finding is especially relevant to Chinese educators because traditional Chinese language classrooms have been teacher-centered and text-based, and the main purpose of teaching is to make students fully understand a prescribed list of texts through teacher explanations; thus, students may find it difficult to develop intrinsic motivation in reading. Recognizing student interests, offering a rationale for reading, and providing choices are promising reading-promotion strategies (Skinner and Belmont, 1993; Reeve, 2002).

Furthermore, intrinsic reading motivation more strongly predicted reading strategy than did extrinsic reading motivation. This result supported Lau and Chan’s (2003) finding that Chinese good readers, who have higher levels of intrinsic reading motivation than poor readers, are more capable of using all reading strategies (especially sophisticated cognitive and metacognitive strategies). Consistently, successful strategy instruction programs have primarily focused on enhancing students’ intrinsic interest in reading (e.g., Guthrie et al., 1996; Guthrie and Wigfield, 2000). This is particularly important for Chinese educators as the traditional methods of Chinese reading instruction are rote learning and drilling, which may cause students to lose intrinsic motivation in reading. Therefore, Chinese educators and researchers should consider redesigning the classroom environment and reading curriculum to help students develop their own interest in reading and strategy learning.

Finally, extrinsic reading motivation did not predict reading amount and even negatively predicted reading achievement when the effect of intrinsic reading motivation was simultaneously tested. This result does not support the argument that extrinsic reading motivation could positively motivate Chinese students (Shih, 2005; Lau and Lee, 2008) and that both intrinsic and extrinsic reading motivation contribute to the amount that students read (Wigfield, 1997; Guthrie et al., 1999). Conversely, this result suggests that extrinsic reading motivation should not be emphasized to motivate low-frequency Chinese students to read more. This view is further supported by the fact that the detrimental effects of extrinsic reading motivation partially neutralized the beneficial effects of intrinsic reading motivation (Schiefele and Löweke, 2017).

### Limitations and Future Research

Although this study evidenced the reliability and validity of the proposed dimensions of intrinsic and extrinsic reading motivation, as well as the relationships among intrinsic reading motivation, extrinsic reading motivation, reading amount, reading strategy, and reading achievement, several limitations should be acknowledged.

First, our research sample was relatively small and was drawn from only two schools in Eastern China, thus limiting the generalizability of these results. Future studies should use nationwide sampling methods or more diverse samples to generate more generalizable results.

Second, reading achievement was assessed using students’ Chinese language grades, which most often reflect students’ ability to memorize the knowledge and explanations of texts provided by teachers. Future research may use measures that are more objective, such as self-constructed or standardized reading comprehension tests, to assess students’ reading achievement. The effects of reading motivation on various reading achievement indicators, such as lower versus higher comprehension levels, should also be evaluated.

Third, the reading strategy construct in the present study included both surface- and deep-level strategies, which may differently affect other reading-related factors. Future studies might distinguish between surface- and deep-level strategies, investigating whether different effects are presented. Furthermore, other methods that assess reading strategies, such as observation and think-aloud, in addition to a self-report inventory, are needed in future research.

Finally, this study did not control for students’ prior reading achievements, which may have inflated the effect of reading motivation on current reading achievement levels. Further research should include students’ past reading achievements in the structural model.

## Data Availability Statement

The raw data supporting the conclusions of this article will be made available by the authors, without undue reservation, to any qualified researcher.

## Ethics Statement

The studies involving human participants were reviewed and approved by the Ethics Committee of Jiangnan University. Written informed consent to participate in this study was provided by the participants’ legal guardian/next of kin.

## Author Contributions

XW designed the study and was responsible for the statistical analyses and the writing of the manuscript. YJ and LJ provided ideas for data analysis and manuscript writing. All authors read the manuscript and approved it for publication.

## Conflict of Interest

The authors declare that the research was conducted in the absence of any commercial or financial relationships that could be construed as a potential conflict of interest.
