# Supplementary material for: Reading Amount and Reading Strategy as Mediators of the Effects of Intrinsic and Extrinsic Reading Motivation on Reading Achievement
Source: Front Psychol. 2020 Oct 27;11:586346. doi: 10.3389/fpsyg.2020.586346 (PMC7652739; doi:10.3389/fpsyg.2020.586346)
Supplement: Supplementary file 1 [file Table_1.DOC]

**Appendix A**

**The Abbreviated Version of Motivation for Reading Questionnaire**

|  | | *very different from me* | *a little different from me* | *a little like me* | *a lot like me* |
| --- | --- | --- | --- | --- | --- |
| *Challenge* | | | | | |
| 1. | I like hard, challenging books. | 1 | 2 | 3 | 4 |
| 2. | I like it when the questions in books make me think. | 1 | 2 | 3 | 4 |
| 3. | I usually learn difficult things by reading. | 1 | 2 | 3 | 4 |
| 4. | If the project is interesting, I can read difficult material. | 1 | 2 | 3 | 4 |
| 5. | If a book is interesting I don’t care how hard it is to read. | 1 | 2 | 3 | 4 |
| *Curiosity* | |  |  |  |  |
| 6. | If the teacher discusses something interesting I might read more about it. | 1 | 2 | 3 | 4 |
| 7. | I read about my hobbies to learn more about them. | 1 | 2 | 3 | 4 |
| 8. | I read to learn new information about topics that interest me. | 1 | 2 | 3 | 4 |
| 9. | I like to read about new things. | 1 | 2 | 3 | 4 |
| 10. | If I am reading about an interesting topic I sometimes lose track of time. | 1 | 2 | 3 | 4 |
| *Involvement* | | | | | |
| 11. | I make pictures in my mind when I read. | 1 | 2 | 3 | 4 |
| 12. | I feel like I make friends with people in good books. | 1 | 2 | 3 | 4 |
| 13. | I like mysteries. | 1 | 2 | 3 | 4 |
| 14. | I enjoy a long, involved story or fiction book. | 1 | 2 | 3 | 4 |
| 15. | I read a lot of adventure stories. | 1 | 2 | 3 | 4 |
| *Importance* | | | | | |
| 16. | It is very important to me to be a good reader. | 1 | 2 | 3 | 4 |
| 17. | In comparison to other activities I do, it is very important to me to be a good reader. | 1 | 2 | 3 | 4 |
| *Recognition* | | | | | |
| 18. | My friends sometimes tell me I am a good reader. | 1 | 2 | 3 | 4 |
| 19. | I like hearing the teacher say I read well. | 1 | 2 | 3 | 4 |
| 20. | I am happy when someone recognizes my reading. | 1 | 2 | 3 | 4 |
| 21. | My parents often tell me what a good job I am doing in reading. | 1 | 2 | 3 | 4 |
| 22. | I like to get compliments for my reading. | 1 | 2 | 3 | 4 |
| *Grades* | | | | | |
| 23. | I look forward to finding out my reading grade. | 1 | 2 | 3 | 4 |
| 24. | Grades are a good way to see how well you are doing in reading. | 1 | 2 | 3 | 4 |
| 25. | I read to improve my grades. | 1 | 2 | 3 | 4 |
| 26. | My parents ask me about my reading grade. | 1 | 2 | 3 | 4 |
| *Competition* | | | | | |
| 27. | I like being the only one who knows an answer in something we read. | 1 | 2 | 3 | 4 |
| 28. | I try to get more answers right than my friends. | 1 | 2 | 3 | 4 |
| 29. | I like to finish my reading before other students. | 1 | 2 | 3 | 4 |
| 30. | I am willing to work hard to read better than my friends. | 1 | 2 | 3 | 4 |
| *Social* | | | | | |
| 31. | I visit the library often with my family. | 1 | 2 | 3 | 4 |
| 32. | I often read to my brother or my sister. | 1 | 2 | 3 | 4 |
| 33. | I sometimes read to my parents. | 1 | 2 | 3 | 4 |
| 34. | My friends and I like to trade things to read. | 1 | 2 | 3 | 4 |
| 35. | I talk to my friends about what I am reading. | 1 | 2 | 3 | 4 |
| 36. | I like to help my friends with their schoolwork in reading. | 1 | 2 | 3 | 4 |
| 37. | I like to tell my family about what I am reading. | 1 | 2 | 3 | 4 |
| *Compliance* | | | | | |
| 38. | I always do my reading work exactly as the teacher wants it. | 1 | 2 | 3 | 4 |
| 39. | Finishing every reading assignment is very important to me. | 1 | 2 | 3 | 4 |
| 40. | I always try to finish my reading on time. | 1 | 2 | 3 | 4 |
