# Supplementary material for: Reading Amount and Reading Strategy as Mediators of the Effects of Intrinsic and Extrinsic Reading Motivation on Reading Achievement
Source: Front Psychol. 2020 Oct 27;11:586346. doi: 10.3389/fpsyg.2020.586346 (PMC7652739; doi:10.3389/fpsyg.2020.586346)
Supplement: Supplementary file 2 [file Table_2.DOC]

**Appendix B**

**The Survey of Adolescent Reading Attitudes**

| *How do you feel about…* | | *Very bad* |  |  |  |  | *Very good* |
| --- | --- | --- | --- | --- | --- | --- | --- |
| *Academic digital* | | | | | | | |
| 1. | reading news online for class? | 1 | 2 | 3 | 4 | 5 | 6 |
| 5. | reading online for a class? | 1 | 2 | 3 | 4 | 5 | 6 |
| 7. | reading a book online for a class? | 1 | 2 | 3 | 4 | 5 | 6 |
| 12. | working on an internet project with classmates? | 1 | 2 | 3 | 4 | 5 | 6 |
| 16. | looking up information online for a class? | 1 | 2 | 3 | 4 | 5 | 6 |
| *Recreational print* | | | | | | | |
| 2. | reading a book in your free time? | 1 | 2 | 3 | 4 | 5 | 6 |
| 8. | talking with friends about something you’ve been reading in your free time? | 1 | 2 | 3 | 4 | 5 | 6 |
| 9. | getting a book or a magazine for a present? | 1 | 2 | 3 | 4 | 5 | 6 |
| 11. | reading a book for fun on a rainy Saturday? | 1 | 2 | 3 | 4 | 5 | 6 |
| 13. | reading anything printed (book, magazines, comic books, etc.) in your free time? | 1 | 2 | 3 | 4 | 5 | 6 |
| *Academic print* | | | | | | | |
| 3. | doing research using encyclopedias (or other books) for a class? | 1 | 2 | 3 | 4 | 5 | 6 |
| 6. | reading a textbook? | 1 | 2 | 3 | 4 | 5 | 6 |
| 14. | using a dictionary for class? | 1 | 2 | 3 | 4 | 5 | 6 |
| 17. | reading a newspaper or a magazine for a class? | 1 | 2 | 3 | 4 | 5 | 6 |
| 18. | reading a novel for class? | 1 | 2 | 3 | 4 | 5 | 6 |
| *Recreational digital* | | | | | | | |
| 4. | instant messaging or e-mailing friends in your free time? | 1 | 2 | 3 | 4 | 5 | 6 |
| 10. | texting friends in your free time? | 1 | 2 | 3 | 4 | 5 | 6 |
| 15. | using social media like Weibo or WeChat in your free time? | 1 | 2 | 3 | 4 | 5 | 6 |
