# Supplementary material for: Reading Amount and Reading Strategy as Mediators of the Effects of Intrinsic and Extrinsic Reading Motivation on Reading Achievement
Source: Front Psychol. 2020 Oct 27;11:586346. doi: 10.3389/fpsyg.2020.586346 (PMC7652739; doi:10.3389/fpsyg.2020.586346)
Supplement: Supplementary file 3 [file Table_3.DOC]

**Appendix C**

**The Reading Amount Scale**

|  |  | 1 | 2 | 3 | 4 |
| --- | --- | --- | --- | --- | --- |
| 1. | How many books did you read for interest during the previous month? | *0 books* | *1–2 books* | *3–4 books* | *More than 5 books* |
|  |  |  |  |  |  |
| 2. | How often do you read for interest? | *Almost never* | *Once a month* | *Once a week* | *Almost every day* |
|  |  |  |  |  |  |
| 3. | How long do you usually spend reading a book without taking a break when reading for interest? | *5 minutes* | *15 minutes* | *30 minutes* | *60 minutes or more* |

Additional question:

Write down the titles of books (up to a maximum of three) that you had read for interest during the previous month.
