# Supplementary material for: Reading Amount and Reading Strategy as Mediators of the Effects of Intrinsic and Extrinsic Reading Motivation on Reading Achievement
Source: Front Psychol. 2020 Oct 27;11:586346. doi: 10.3389/fpsyg.2020.586346 (PMC7652739; doi:10.3389/fpsyg.2020.586346)
Supplement: Supplementary file 4 [file Table_4.DOC]

**Appendix D**

**The Cognitive Strategy Questionnaire**

| *When I study…* | | *Almost never* | *Sometimes* | *Often* | *Almost always* |
| --- | --- | --- | --- | --- | --- |
| *Memorization* | | | | | |
| 1. | I try to memorize everything that is covered in the text. | 1 | 2 | 3 | 4 |
| 3. | I try to memorize as many details as possible. | 1 | 2 | 3 | 4 |
| 5. | I read the text so many times that I can recite it. | 1 | 2 | 3 | 4 |
| 7. | I read the text over and over again. | 1 | 2 | 3 | 4 |
| *Elaboration* | | | | | |
| 4. | I try to relate new information to prior knowledge acquired in other subjects. | 1 | 2 | 3 | 4 |
| 8. | I figure out how the information might be useful outside school. | 1 | 2 | 3 | 4 |
| 10. | I try to understand the material better by relating it to my own experiences. | 1 | 2 | 3 | 4 |
| 12. | I figure out how the text information fits in with what happens in real life. | 1 | 2 | 3 | 4 |
| *Control* | | | | | |
| 2. | I start by figuring out what exactly I need to learn. | 1 | 2 | 3 | 4 |
| 6. | I check if I understand what I have read. | 1 | 2 | 3 | 4 |
| 9. | I try to figure out which concepts I still haven’t really understood. | 1 | 2 | 3 | 4 |
| 11. | I make sure that I remember the most important points in the text. | 1 | 2 | 3 | 4 |
| 13. | If I don’t understand something, I look for additional information to clarify this. | 1 | 2 | 3 | 4 |
